# Microsecond molecular dynamics simulations revealed the inhibitory potency of amiloride analogs against SARS-CoV-2 E viroporin

**DOI:** 10.5808/gi.21040

**Published:** 2021-12-31

**Authors:** Abdullah All Jaber, Zeshan Mahmud Chowdhury, Arittra Bhattacharjee, Muntahi Mourin, Chaman Ara Keya, Zaied Ahmed Bhuyan

**Affiliations:** 1 Department of Biochemistry and Microbiology, North South University, Bashundhara, Dhaka-1229, Bangladesh; 2 Bioinformatics Division, National Institute of Biotechnology, Ganakbari, Ashulia, Savar, Dhaka-1349, Bangladesh; 3 Department of Microbiology, University of Manitoba, 66 Chancellors Cir, Winnipeg, MB R3T 2N2, Canada

**Keywords:** COVID-19, hexamethylene amiloride, SARS-CoV-2 E, virology, viroporin

## Abstract

Severe acute respiratory syndrome coronavirus 2 (SARS-CoV-2) encodes small envelope protein (E) that plays a major role in viral assembly, release, pathogenesis, and host inflammation. Previous studies demonstrated that pyrazine ring containing amiloride analogs inhibit this protein in different types of coronavirus including SARS-CoV-1 small envelope protein E (SARS-CoV-1 E). SARS-CoV-1 E has 93.42% sequence identity with SARS-CoV-2 E and shared a conserved domain NS3/small envelope protein (NS3_envE). Amiloride analog hexamethylene amiloride (HMA) can inhibit SARS-CoV-1 E. Therefore, we performed molecular docking and dynamics simulations to explore whether amiloride analogs are effective in inhibiting SARS-CoV-2 E. To do so, SARS-CoV-1 E and SARS-CoV-2 E proteins were taken as receptors while HMA and 3-amino-5-(azepan-1-yl)-N-(diaminomethylidene)-6-pyrimidin-5-ylpyrazine-2-carboxamide (3A5NP2C) were selected as ligands. Molecular docking simulation showed higher binding affinity scores of HMA and 3A5NP2C for SARS-CoV-2 E than SARS-CoV-1 E. Moreover, HMA and 3A5NP2C engaged more amino acids in SARS-CoV-2 E. Molecular dynamics simulation for 1 μs (1,000 ns) revealed that these ligands could alter the native structure of the proteins and their flexibility. Our study suggests that suitable amiloride analogs might yield a prospective drug against coronavirus disease 2019.

## Introduction

Severe acute respiratory syndrome coronavirus-2 (SARS-CoV-2), the etiological agent of coronavirus disease 2019 (COVID-19), is a threat to the global public health and economy [1]. SARS-CoV or SARS-CoV-1, which is around 80% genetically similar to SARS-CoV-2, initiated the fatal outbreak of severe acute respiratory syndrome (SARS) in Southeast China, 2002 [2-5]. SARS-CoV-1 and SARS-CoV-2 encode viroporins (VPs) that exhibit ion channel (IC) activity in both host cell and virion. VPs are crucial factors for viral pathogenesis, infection cycle, virion morphogenesis, assembly, and viral release from the host cells [6,7]. Besides SARS-like coronaviruses, other pathogenic human viruses such as human immunodeficiency virus-1 (HIV-1), influenza A virus (IAV), rotavirus also encode VPs [7-10]. VPs induce ion imbalance within host cells and disrupt cellular pathways via various protein-protein interactions [11].

SARS-CoV-1 encodes VPs from small envelope protein E, ORF3a and ORF8a genes. Protein E and ORF3a interact with cellular proteins via PDZ-binding motif and exerts IC activity. These functions are essential for optimal viral replication. However, among these 3 VP-forming genes, protein E was indispensable for viral virulence [12]. Protein E forms calcium ion (Ca^2+^) channels in the endoplas­mic reticulum golgi apparatus intermediate compartment (ERGIC)/Golgi membranes. These ICs alter calcium homeostasis in host cells and activate the cytosolic innate immune signaling receptor NLR family pyrin domain containing 3 inflammasome [13,14]. Absence of protein E attenuates the viral infectious activity by reducing nuclear factor kB mediated inflammation [15]. Therefore, protein E can be a plausible therapeutic target for COVID-19.

Previous studies demonstrated that VP-like prototypic M2 proton selective channel of IAV can be an ideal target for antiviral development [16]. All coronavirus E proteins are assumed to form cation-selective ion channels which participate in viral replication and virus’s life cycle [17,18]. The actions of VPs in SARS-CoV-2 may also be figured out from other CoVs e.g., Middle East respiratory syndrome coronavirus and human coronavirus 229E (HCoV-229E) [5,6,19]. Deletion of E gene from SARS-CoV-1 significantly decreased the viral pathogenesis [15]. Moreover, the usage of the channel blocking compounds such as amiloride analogs had substantially alleviated the viral replications of HCoV-229E, murine hepatitis virus (MHV), and SARS-CoV-1 [20,21]. Here, the amiloride analogs interacted with the E proteins and inhibited the functions of VP [22,23]. These results have raised a possibility to develop a broad spectrum antiviral drug against coronaviruses [20-23]. Aside from coronaviruses, Amiloride derivatives (particularly hexamethylene amiloride [HMA]) were found to be efficient inhibitors of ICs in hepatitis C virus, influenza virus, and HIV-1 [7-9]. Since E gene of SARS-CoV-1 and SARS-CoV-2 are highly identical (Table 1, Supplementary Data 1), implementation of different *in silico* tools can unveil the anti-SARS-CoV-2 E mechanisms of amiloride analogs [24,25].

Amiloride is a pyrazine ring containing compound that inhibits sodium-hydrogen antiporter 1 (NHE-1) and promotes diuresis [26]. Analogs of amiloride such as 5-(N,N-Hexamethylene) amiloride or HMA (PubChem CID: 1794) can inhibit urokinase-type plasminogen activator (uPA) which is an important protease for tumor cell to undergo metastasis [27,28]. HMA has antiviral activities with little K^+^ sparing diuretic effect and 3-Amino-5-(azepan-1-yl)-N-(diaminomethylidene)-6-pyrimidin-5-ylpyrazine-2-carboxamide (3A5NP2C) (PubChem CID: 137348787), a structurally similar compound, has very little cytotoxic properties in human cells [27,29]. In this study, we explored the anti-SARS-CoV-2 E activities of HMA and 3A5NP2C. Through molecular docking and molecular dynamics simulations, we showed that these small molecules could bind and alter the structure of SARS-CoV-2 E in golgi membrane lipid bilayer. Our study suggests that amiloride analogs could be a druggable compound for COVID-19.

## Methods

### Characterization of SARS-CoV-2 E VP

For characterization of the SARS-CoV-2 E, the E protein (accession: YP_009724392.1) went under Protein Basic Local Alignment Search Tool (BLASTp) in National Center for Biotechnology Information (NCBI) database. To find homologous sequences in other CoVs, SARS-CoV-2 was excluded during this BLASTp. Top 4 hits in BLAST were taken for characterization. Afterward, E protein of SARS-CoV-1 GD01 (accession: AAP51230.1) was also included. The protein sequences were uploaded in ProtParam, Pfam, and THMM to analyze their physicochemical properties, domains, and transmembrane helices [30-32]. Their phylogenetic characterization was conducted by CLC Drug Discovery Workbench 3.0 using default parameters (https://digitalinsights.qiagen.com/).

### Three-dimensional structure generations of E proteins

E proteins of SARS-CoV-1 and SARS-CoV-2 were generated via Robetta (https://robetta.bakerlab.org/) [33]. The structures were refined with three-dimensional refine and Galaxy Refine [34,35]. The qualities of the generated structures were assessed using SWISS structure assessment [36].

### Pharmacokinetic property exploration of the ligands and preparation for molecular docking

The molecules were selected after going through literature [27,28]. The cannonical smiles of 5-(N,N-hexamethylene) amiloride or HMA and 3A5NP2C were uploaded in SWISS-ADME for exploring the pharmacokinetic properties (absorption, distribution, metabolism, excretion) [37]. The canonical SMILES (Simplified molecular-input line-entry system) were collected from PubChem database and transferred into protein data bank file via Online SMILES Translator and Structure File Generator (https://cactus.nci.nih.gov/translate/) [38].

### Molecular docking and dynamic simulations

The selected ligands went under blind molecular docking simulation against SARS-CoV-1 and SARS-CoV-2 E proteins via AutoDock tools 1.5.6 [39]. The ligand-receptor interactions between drug-receptor complexes were visualized by BIOVIA Discovery Studio (http://www.discover.3ds.com) and PyMOL Molecular Graphics System, Version 2.3.3 Schrödinger, LLC [40] and UCSF Chimera [41]. The dynamics and stability of SARS-CoV-1 and SARS-CoV-2 E proteins with amiloride analogs bound complexes were compared by carrying out MD simulations. For MD simulations, the drug-bound complexes were embedded in a lipid bilayer membrane composed of 36% phosphatidylcholine, 21% phosphatidylethanolamine, 21% cholesterol, 6% phosphatidylserine. This lipid bilayer mimics the lipid composition of gogli complex [42]. The SARS-CoV-1 VPs generally target golgi apparatus [43]. The total system consisted of about 120,000 atoms in an orthorhombic simulation cell with a free KCl concentration of 150 mM. Equilibrium MD simulations were performed after energy minimization and 1,000 ns of equilibration with position restraints. The protein, water, and lipid components were energy minimized using Charmm-Gui Bilayer Builder [44] and Gromacs 2019.2 [45,46]. All simulations were carried out under periodic boundary conditions at constant temperature (T = 310°K) and pressure (P = 1 bar). Then the root mean square deviation (RMSD) and Root Mean Square Fluctuation (RMSF) of C-α carbon from wild type apo-SARS-CoV-1 E and apo-SARS-CoV-2 E with ligand bound complexes were analyzed by g_rms, g_rmsf tools.

## Results

### SARS-CoV-2 E protein contains relatively higher instability index

According to NCBI BLASTp, SARS-CoV-1 and SARS-CoV-2 E proteins share 93.42% sequence identity. The selected CoV E protein contains nearly same numbers of amino acids outside and inside of the transmembrane. Their grand average of hydropathicity values are not significantly diverse. However, SARS-CoV-2 E has a higher theoretical pI (isoelectric point) and instability index than average value. Only Bat SARS-like coronavirus has this type of properties.

**Fig. 1.**
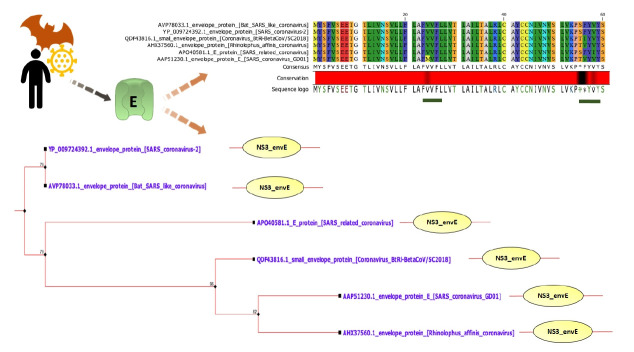
E protein is highly conserved among SARS-CoV-2 and SARS-CoV-1. Only black regions (underlined with green bars) are variable. All of the E proteins have NS3/small envelope protein E domain (NS3_envE). According to the phylogenetic tree, SARS-CoV-2 E protein is mostly closed to bat SARS-like coronavirus E protein. E, envelope; SARS-CoV, severe acute respiratory syndrome coronavirus.

All of the E proteins have Non-structural protein NS3/Small envelope protein E domain (Fig. 1). Some insignificant domains such as Ellis van Creveld protein 2 like protein and FAM163 family were found in SARS-CoV-2 E. This insignificant FAM163 family domain was absent in SARS-CoV-1 E (Supplementary Data 1).

### SARS-CoV-2 E protein demonstrated higher binding affinity for the ligands than SARS-CoV-1 E

**Fig. 2.**
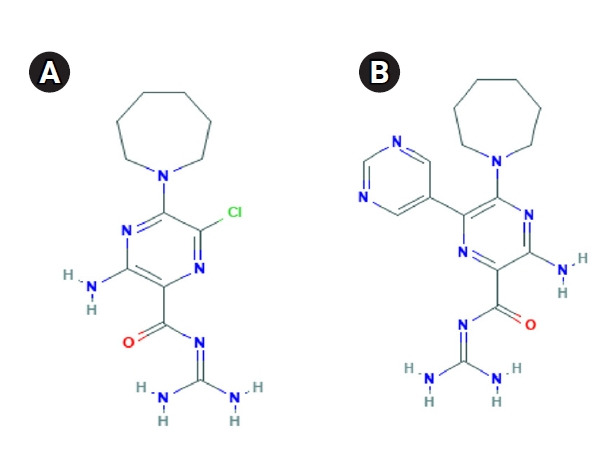
Selected compound for E protein inhibition. (A) 5-(N,N-hexamethylene) amiloride is a well-known coronavirus viroporin inhibitor. (B) Structurally similar 3-amino-5-(azepan-1-yl)-N-(diaminomethylidene)-6-pyrimidin-5-ylpyrazine-2-carboxamide (3A5NP2C). This small molecule has anticancer activity. E, envelope.

The E proteins of SARS-CoV-1 and SARS-CoV-2 went under homology modeling and their structures were refined. More than 97% amino acids were in favored region of Ramachandran plot. Details about the structural qualities are given in Supplementary Data 2. To target SARS-CoV-2 E, anticancer amiloride analogs, i.e., HMA and 3A5NP2C were used in this study (Fig. 2). Their physicochemical and pharmacokinetic properties are given in (Table 2) and Supplementary Data 2. They have similar bioavailability. HMA might inhibit CYP1A2 and CYP2C19. However, this characteristic was absent in 3A5NP2C. SARS-CoV-1 E demonstrated ‒5.5 kcal/mol and ‒5.8 kcal/mol binding affinity scores with HMA and 3A5NP2C respectively. Whereas HMA and 3A5NP2C interacted with SARS-CoV-2 E with ‒7.3 kcal/mol and ‒7.1 kcal/mol binding affinity scores, respectively (Table 2).

**Fig. 3.**
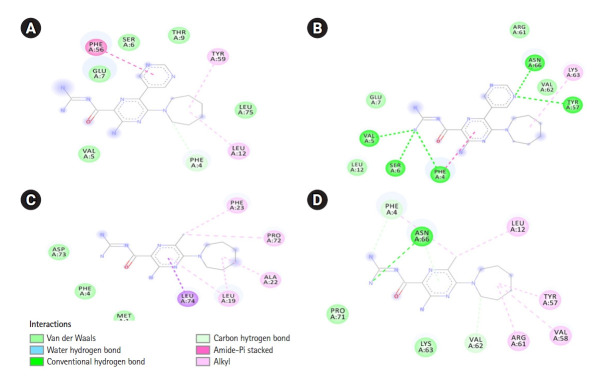
Interactions between 3A5NP2C with SARS-CoV-1 E (A) and SARS-CoV-2 E (B). 5-(N,N-Hexamethylene) amiloride interactions with SARS-CoV-1 E (C) and SARS-CoV-2 E viroporins (D). 3A5NP2C, 3-amino-5-(azepan-1-yl)-N-(diaminomethylidene)-6-pyrimidin-5-ylpyrazine-2-carboxamide; E, envelope; SARS-CoV, severe acute respiratory syndrome coronavirus.

Only Phe 4 of SARS-CoV-1 E interacted with both HMA and 3A5NP2C. Whereas six common residues of SARS-CoV-2 protein E Phe 4, Asn 66, Leu 12, Tyr 57, Val 62, Lys 63 participated in ligand-receptor interactions (Fig. 3). Moreover, HMA and 3A5NP2C interacted via H-bonds with SARS-CoV-2 E which was not observed in SARS-CoV-1 E. The pyrazine ring, which is essential for the structure activity relationship of amiloride, showed various types of interactions. The extra pyrimidine group of 3A5NP2C engaged more amino acids in the receptors.

### HMA and 3A5NP2C altered the wild type E proteins structures

**Fig. 4.**
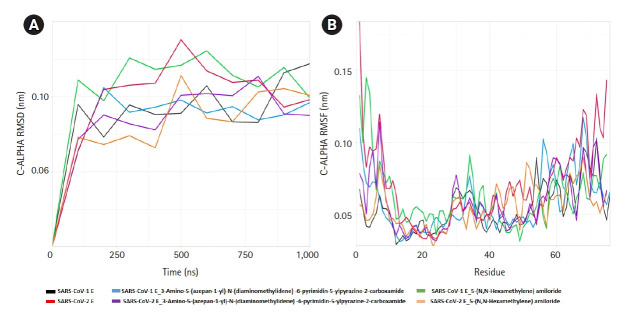
Molecular dynamics simulation of amiloride analogs and the receptors. The root mean square deviation (RMSD) (A) and root mean square fluctuation (RMSF) (B) of SARS-CoV-1 and 2 E proteins with and without amiloride analogs. SARS-CoV, severe acute respiratory syndrome coronavirus; E, envelope.

The ligand-receptor complexes went under 1 ms simulation or 1,000 ns MD simulation. The ligand bound and ligand-free E proteins deviated in same pattern for less than 100 ns and after that their RMSD values were different (Fig. 4). Ligand-receptor complexes also showed altered mobility, especially in the cytoplasmic, transmembrane, and non-cytoplasmic regions. SARS-CoV-2-E-3A5NP2C complex also demonstrated different RMSD value at 100ns whereas SARS-CoV-2-E–HMA showed less deviations. On the other hand, HMA reduced more fluctuation than 3A5NP2C in these domains of SARS-CoV-2 E proteins. During the simulation, the RMSD of SARS-CoV-1 E (black line) and SARS-CoV-1-E-HMA (green line) did not show equal overlapping value after few seconds. This indicated that the bonded drug altered the wild type conformations hence it could not execute the IC activities [22]. Similar patterns were also observed between SARS-CoV-2 E and drug bound SARS-CoV-2 E. RMSF analysis showed that these compounds reduced the mobility of N and C terminal region of SARS-CoV-2 E (Fig. 4).

**Fig. 5.**
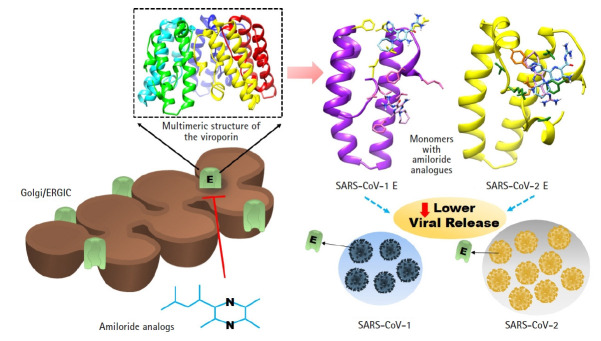
The schematic representation of the whole study. E, envelope; SARS-CoV, severe acute respiratory syndrome coronavirus; ERGIC, Endoplasmic Reticulum Golgi Apparatus Intermediate Compartment.

The schematic representation of the whole study is given in Fig. 5.

## Discussion

VPs are involved in viral assembly and pathogenesis, which promotes ion imbalance within host cells, disrupting cellular pathways [11,43,47]. Therefore, impeding their activity by specific drugs offers promising antiviral therapeutics [6,48]. Coronaviruses, including SARS-CoV-1, express small envelope (E) protein that forms VPs. These VPs act as cation-selective ion channels in lipid bilayers that are able to pump Na^+^ and K^+^ ions [49]. The transmembrane α-helical domain of E forming multimeric α-helical bundle is responsible for the IC activity [50]. Similar to SARS-CoV-1, SARS-CoV-2 also encodes VP forming E protein [51,52]. This E protein is 93.42% identical with SARS-CoV-1 E. SARS-CoV-2 E has a conserved NS3/Small envelope protein E domain (NS3_envE) that triggers inflammation in host cells (Fig. 1) [13].

Previous reports showed that NHE-1 blocker HMA inhibits HCoV-229E and MHV E protein IC conductance in lipid bilayers [21]. Functions of SARS-CoV-1 E got significantly reduced by HMA in human embryonic kidney 293 cells [22]. Moreover, HMA and 3A5NP2C have anticancer properties [27,28]. According to SWISS-ADME, these molecules are impenetrable through blood brain barrier and can circulate in the body with 0.55 bioavailability score. 3A5NP2C drug compound has lower gastrointestinal absorption than HMA. However, HMA might act as an inhibitory substrate of CYP1A2 and CYP2C19 enzyme. Specific modification in the side chain of amiloride analogs might make the compound a potential broad spectrum anti-microbial compound with safer ADMET properties and efficacy. Hence, further studies may contribute to the structural modification of these analogs devising a safer and more effective antiviral [53].

In this study, we have found that the structural characteristics of HMA and similar compound 3A5NP2C exhibited more binding affinity score with SARS-CoV-2 E than SARS-CoV-1 E. Phe 4 of SARS-CoV-1 E was the common interacted residue for both HMA and 3A5NP2C whereas SARS-CoV-2 E interacted via Phe 4, Asn 66, Leu 12, Tyr 57, Val 62, Lys 63. This indicated that these ligands could bind to both inside (amino acid number: 1‒11), outside (amino acid number: 35‒75) and within transmembrane helix (amino acid number: 12‒34) regions of SARS-CoV-2 E (Table 1).

The E proteins and drug-receptor complexes were placed in a Golgi lipid bilayer model for MD simulations. SARS-CoV-2 E showed higher mobility and deviations in bilayer than SARS-CoV-1 E. Especially the outside N and C terminal regions of the protein were more mobile than SARS-CoV-1 E. Since, SARS-CoV-2 can replicate 3 times faster than SARS-CoV-1; hence, it can release/shed more viruses from the host cells [54]. Whether this enhanced mobility and instability of SARS-CoV-2 E are contributing in higher viral release is further needed to be explored (Table 1, Fig. 4). Here, the SARS-CoV-1 E and SARS-CoV-1-E-HMA complex were run as control since HMA can inhibit the VP activities. The differences between RMSD and RMSF of the ligand-bound and ligand-free SARS-CoV-1 E indicated that these compounds interfered with the normal physiology of the viral protein. The same patterns were also observed for ligand-bound and ligand-free SARS-CoV-2 E proteins. The flexibility of the proteins was altered in cytoplasmic, non-cytoplasmic and transmembrane regions. These flexibilities are critical for viral physiology [43]. MD simulation revealed that this region would gain rigidity which strongly supports that the viral physiology will be hindered extremely [55]. Hence, these deviations and alterations of the protein E strongly suggest that these amiloride analogs will disrupt the cation-selective IC activities.

Amiloride and HMA are well-studied compounds; therefore, in the near future, their antiviral activity against SARS-CoV-2 can be evaluated to develop new treatments. However, amiloride has some rare adverse effects and side effects. The most dangerous effects include hyperkalaemia [56]. Therefore, similar to nafamostat mesylate, serum potassium values should be monitored carefully in COVID-19 patients after the administration of amiloride analogs [57]. Although HMA has a lower K^+^ sparing diuretic effect than amiloride, these drugs might show side effects by hindering the general physiology of uPA [27,28].

Several amiloride analogs can inhibit the functions of coronavirus VPs. Our study strongly suggests their antiviral activities against SARS-CoV-2. Further *in vitro* screening and *in vivo* experiments are necessary to consider amiloride analogs as a prospective drug against this virus.

## Figures and Tables

**Table 1.** Transmembrane positions of amino acids, theoretical pI (isoelectric point), instability index, aliphatic index, and GRAVY of E proteins in several coronaviruses related to SARS-CoV-2

Serial No.	Virus	NCBI accession No. for E protein	No. of AA's	Outside	Inside	Theoretical pI	Instability Index	Aliphatic Index	GRAVY
1	Bat SARS-like coronavirus	AVP78033.1	75	1‒11	35‒75	8.57	38.68	144	1.128
2	SARS-related coronavirus	APO40581.1	76	1‒11	35‒76	7.69	35.26	144.74	1.129
3	Coronavirus BtRl-BetaCoV/SC2018	QDF43816.1	76	1‒11	35‒76	6.01	31.47	147.24	1.145
4	Rhinolophus affinis coronavirus	AHX37560.1	76	1‒11	35‒76	6.01	33.02	145.92	1.176
5	SARS-CoV-2	YP_009724392.1	75	1‒11	35‒76	8.57	38.68	144	1.128
6	SARS coronavirus GD01	AAP51230.1	76	1‒11	35‒75	6.01	30.48	142.11	1.111
Average	-	-	75.67	-	-	7.12	33.782	144.67	1.14

GRAVY, grand average of hydropathicity; SARS-CoV-2, severe acute respiratory syndrome coronavirus 2.

**Table 2.** Major physiochemical and pharmacokinetic characteristics of the selected anticancer amiloride analogs

Characteristic	5-(N,N-Hexamethylene) amiloride (HMA)	3-Amino-5-(azepan-1-yl)-N-(diaminomethylidene)-6-pyrimidin-5-ylpyrazine-2-carboxamide (3A5NP2C)
Molecular weight	311.77	355.4
H-bond acceptors	3	4
H-bond donors	4	6
GI absorption	High	Low
BBB permeant	No	No
Inhibitory substrate of:	CYP1A2, CYP2C19	No data available
Bioavailability score	0.55	0.55
Binding affinity score (SARS-CoV-1 E) (kcal/mol)	‒5.5	‒5.8
Binding affinity score (SARS-CoV-2 E) (kcal/mol)	‒7.3	‒7.1

GI, gastrointestinal absorption; BBB, blood brain barrier; SARS-CoV, severe acute respiratory syndrome coronavirus; E, envelope protein.

## References

[1] Keni R, Alexander A, Nayak PG, Mudgal J, Nandakumar K (2020). COVID-19: emergence, spread, possible treatments, and global burden. Front Public Health.

[2] Lau SK, Woo PC, Li KS, Huang Y, Tsoi HW, Wong BH (2005). Severe acute respiratory syndrome coronavirus-like virus in Chinese horseshoe bats. Proc Natl Acad Sci U S A.

[3] Debbink K, Agnihothram S, Gralinski LE, Plante JA (2015). A SARS-like cluster of circulating bat coronaviruses shows potential for human emergence. Nat Med.

[4] Zaki AM, van Boheemen S, Bestebroer TM, Osterhaus AD, Fouchier RA (2012). Isolation of a novel coronavirus from a man with pneumonia in Saudi Arabia. N Engl J Med.

[5] Zhu Z, Lian X, Su X, Wu W, Marraro GA, Zeng Y (2020). From SARS and MERS to COVID-19: a brief summary and comparison of severe acute respiratory infections caused by three highly pathogenic human coronaviruses. Respir Res.

[6] Castano-Rodriguez C, Honrubia JM, Gutierrez-Alvarez J, DeDiego ML, Nieto-Torres JL, Jimenez-Guardeno JM (2018). Role of severe acute respiratory syndrome coronavirus viroporins E, 3a, and 8a in replication and pathogenesis. mBio.

[7] Ewart GD, Sutherland T, Gage PW, Cox GB (1996). The Vpu protein of human immunodeficiency virus type 1 forms cation-selective ion channels. J Virol.

[8] Pinto LH, Holsinger LJ, Lamb RA (1992). Influenza virus M2 protein has ion channel activity. Cell.

[9] Pavlovic D, Neville DC, Argaud O, Blumberg B, Dwek RA, Fischer WB (2003). The hepatitis C virus p7 protein forms an ion channel that is inhibited by long-alkyl-chain iminosugar derivatives. Proc Natl Acad Sci U S A.

[10] Hyser JM, Collinson-Pautz MR, Utama B, Estes MK (2010). Rotavirus disrupts calcium homeostasis by NSP4 viroporin activity. mBio.

[11] Hover S, Foster B, Barr JN, Mankouri J (2017). Viral dependence on cellular ion channels: an emerging anti-viral target?. J Gen Virol.

[12] Verdia-Baguena C, Nieto-Torres JL, Alcaraz A, DeDiego ML, Torres J, Aguilella VM (2012). Coronavirus E protein forms ion channels with functionally and structurally-involved membrane lipids. Virology.

[13] Nieto-Torres JL, Verdia-Baguena C, Jimenez-Guardeno JM, Regla-Nava JA, Castano-Rodriguez C, Fernandez-Delgado R (2015). Severe acute respiratory syndrome coronavirus E protein transports calcium ions and activates the NLRP3 inflammasome. Virology.

[14] Shah A (2020). Novel coronavirus-induced NLRP3 inflammasome activation: a potential drug target in the treatment of COVID-19. Front Immunol.

[15] DeDiego ML, Nieto-Torres JL, Regla-Nava JA, Jimenez-Guardeno JM, Fernandez-Delgado R, Fett C (2014). Inhibition of NF-kappaB-mediated inflammation in severe acute respiratory syndrome coronavirus-infected mice increases survival. J Virol.

[16] Scott C, Kankanala J, Foster TL, Goldhill DH, Bao P, Simmons K (2020). Site-directed M2 proton channel inhibitors enable synergistic combination therapy for rimantadine-resistant pandemic influenza. PLoS Pathog.

[17] Tong TR (2009). Therapies for coronaviruses. Part 2: Inhibitors of intracellular life cycle. Expert Opin Ther Pat.

[18] Pillaiyar T, Meenakshisundaram S, Manickam M (2020). Recent discovery and development of inhibitors targeting coronaviruses. Drug Discov Today.

[19] Coronaviridae Study Group of the International Committee on Taxonomy of Viruses (2020). The species Severe acute respiratory syndrome-related coronavirus: classifying 2019-nCoV and naming it SARS-CoV-2. Nat Microbiol.

[20] Li S, Yuan L, Dai G, Chen RA, Liu DX, Fung TS (2019). Regulation of the ER stress response by the Ion channel activity of the infectious bronchitis coronavirus envelope protein modulates virion release, apoptosis, viral fitness, and pathogenesis. Front Microbiol.

[21] Wilson L, Gage P, Ewart G (2006). Hexamethylene amiloride blocks E protein ion channels and inhibits coronavirus replication. Virology.

[22] Pervushin K, Tan E, Parthasarathy K, Lin X, Jiang FL, Yu D (2009). Structure and inhibition of the SARS coronavirus envelope protein ion channel. PLoS Pathog.

[23] Silva LR, da Silva Santos-Junior PF, de Andrade Brandao J, Anderson L, Bassi EJ, Xavier de Araujo-Junior J (2020). Druggable targets from coronaviruses for designing new antiviral drugs. Bioorg Med Chem.

[24] Bhattacharjee A, Hossain MU, Chowdhury ZM, Rahman SM, Bhuyan ZA, Salimullah M (2021). Insight of druggable cannabinoids against estrogen receptor beta in breast cancer. J Biomol Struct Dyn.

[25] Hossain MU, Bhattacharjee A, Emon MT, Chowdhury ZM, Mosaib MG, Mourin M (2021). Recognition of plausible therapeutic agents to combat COVID-19: an omics data based combined approach. Gene.

[26] Lant AF, Smith AJ, Wilson GM (1969). Clinical evaluation of amiloride, a potassium-sparing diuretic. Clin Pharmacol Ther.

[27] Buckley BJ, Aboelela A, Minaei E, Jiang LX, Xu Z, Ali U (2018). 6-Substituted hexamethylene amiloride (HMA) derivatives as potent and selective inhibitors of the human urokinase plasminogen activator for use in cancer. J Med Chem.

[28] Matthews H, Ranson M, Tyndall JD, Kelso MJ (2011). Synthesis and preliminary evaluation of amiloride analogs as inhibitors of the urokinase-type plasminogen activator (uPA). Bioorg Med Chem Lett.

[29] Scott C, Griffin S (2015). Viroporins: structure, function and potential as antiviral targets. J Gen Virol.

[30] Gasteiger E, Hoogland C, Gattiker A, Duvaud S, Wilkins MR, Appel RD, Walker JM (2005). The Proteomics Protocols Handbook.

[31] El-Gebali S, Mistry J, Bateman A, Eddy SR, Luciani A, Potter SC (2019). The Pfam protein families database in 2019. Nucleic Acids Res.

[32] Krogh A, Larsson B, von Heijne G, Sonnhammer EL (2001). Predicting transmembrane protein topology with a hidden Markov model: application to complete genomes. J Mol Biol.

[33] Kim DE, Chivian D, Baker D (2004). Protein structure prediction and analysis using the Robetta server. Nucleic Acids Res.

[34] Bhattacharya D, Nowotny J, Cao R, Cheng J (2016). 3Drefine: an interactive web server for efficient protein structure refinement. Nucleic Acids Res.

[35] Ko J, Park H, Heo L, Seok C (2012). GalaxyWEB server for protein structure prediction and refinement. Nucleic Acids Res.

[36] Waterhouse A, Bertoni M, Bienert S, Studer G, Tauriello G, Gumienny R (2018). SWISS-MODEL: homology modelling of protein structures and complexes. Nucleic Acids Res.

[37] Daina A, Michielin O, Zoete V (2017). SwissADME: a free web tool to evaluate pharmacokinetics, drug-likeness and medicinal chemistry friendliness of small molecules. Sci Rep.

[38] Kim S, Thiessen PA, Bolton EE, Chen J, Fu G, Gindulyte A (2016). PubChem substance and compound databases. Nucleic Acids Res.

[39] Trott O, Olson AJ (2010). AutoDock Vina: improving the speed and accuracy of docking with a new scoring function, efficient optimization, and multithreading. J Comput Chem.

[40] DeLano WL Pymol: an open-source molecular graphics tool. Collaborative Computational Project No. 4, 2002. http://www.ccp4.ac.uk/newsletters/newsletter40/11_pymol.pdf.

[41] Pettersen EF, Goddard TD, Huang CC, Couch GS, Greenblatt DM, Meng EC (2004). UCSF Chimera: a visualization system for exploratory research and analysis. J Comput Chem.

[42] Casares D, Escriba PV, Rossello CA (2019). Membrane Lipid Composition: effect on Membrane and Organelle Structure, Function and Compartmentalization and Therapeutic Avenues. Int J Mol Sci.

[43] Nieva JL, Madan V, Carrasco L (2012). Viroporins: structure and biological functions. Nat Rev Microbiol.

[44] Jo S, Kim T, Iyer VG, Im W (2008). CHARMM-GUI: a web-based graphical user interface for CHARMM. J Comput Chem.

[45] Hess B, Kutzner C, van der Spoel D, Lindahl E (2008). GROMACS 4: algorithms for highly efficient, load-balanced, and scalable molecular simulation. J Chem Theory Comput.

[46] Kutzner C, Pall S, Fechner M, Esztermann A, de Groot BL, Grubmuller H (2019). More bang for your buck: Improved use of GPU nodes for GROMACS 2018. J Comput Chem.

[47] V'Kovski P, Kratzel A, Steiner S, Stalder H, Thiel V (2021). Coronavirus biology and replication: implications for SARS-CoV-2. Nat Rev Microbiol.

[48] Rehman M, Tauseef I, Aalia B, Shah SH, Junaid M, Haleem KS (2020). Therapeutic and vaccine strategies against SARS-CoV-2: past, present and future. Future Virol.

[49] Wilson L, McKinlay C, Gage P, Ewart G (2004). SARS coronavirus E protein forms cation-selective ion channels. Virology.

[50] Parthasarathy K, Ng L, Lin X, Liu DX, Pervushin K, Gong X (2008). Structural flexibility of the pentameric SARS coronavirus envelope protein ion channel. Biophys J.

[51] Schoeman D, Fielding BC (2019). Coronavirus envelope protein: current knowledge. Virol J.

[52] Khailany RA, Safdar M, Ozaslan M (2020). Genomic characterization of a novel SARS-CoV-2. Gene Rep.

[53] Mourin M, Bhattacharjee A, Wai A, Hausner G, O'Neil J, Dibrov P (2021). Pharmacophore-based screening and modification of amiloride analogs for targeting the NhaP-type cation-proton antiporter in Vibrio cholerae. Can J Microbiol.

[54] Chu H, Chan JF, Wang Y, Yuen TT, Chai Y, Hou Y (2020). Comparative replication and immune activation profiles of SARS-CoV-2 and SARS-CoV in human lungs: an ex vivo study with implications for the pathogenesis of COVID-19. Clin Infect Dis.

[55] Salsbury FR (2010). Molecular dynamics simulations of protein dynamics and their relevance to drug discovery. Curr Opin Pharmacol.

[56] Sun Q, Sever P (2020). Amiloride: a review. J Renin Angiotensin Aldosterone Syst.

[57] Okajima M, Takahashi Y, Kaji T, Ogawa N, Mouri H (2020). Nafamostat mesylate-induced hyperkalemia in critically ill patients with COVID-19: four case reports. World J Clin Cases.

