# Supplementary material for: Microsecond molecular dynamics simulations revealed the inhibitory potency of amiloride analogs against SARS-CoV-2 E viroporin
Source: Genomics Inform. 2021 Dec 31;19(4):e48. doi: 10.5808/gi.21040 (PMC8752979; doi:10.5808/gi.21040)
Supplement: Supplementary Data S1. [file gi-21040suppl1.pdf]

Supplementary File 1. Physical and Chemical properties, Pfam-A Matches with Structural Assessment of different E protein

Different physical and chemical properties

Serial No.	Name of the Virus	Accession no. for E protein	No. of AA's	Theoretical pI	Instability Index	Aliphatic Index	GRAVY
1	Bat SARS-like coronavirus	AVP78033.1	75	8.57	38.68	144	1.128
2	Severe acute respiratory syndrome-related coronavirus	APO40581.1	76	7.69	35.26	144.74	1.129
3	Coronavirus BtRl-BetaCoV/SC2018	QDF43816.1	76	6.01	31.47	147.24	1.145
4	Rhinolophus affinis coronavirus	AHX37560.1	76	6.01	33.02	145.92	1.176
5	Severe acute respiratory syndrome coronavirus 2	YP_009724392.1	75	8.57	38.68	144	1.128
6	SARS coronavirus GD01	AAP51230.1	76	6.01	30.48	142.11	1.111
**Average**			75.67	7.12	33.782	144.67	1.14

Significant Pfam-A Matches:

Sl. No.	Name of the Virus	Accession No.	Pfam-A Matches	Family	Description	HMM		Envelope				
						**From**	**To**	**Start**	**End**	**HMM length**	**Bit score**	**E-value**
1	Bat SARS-like coronavirus	AVP78033.1	**Significant**	NS3_envE	Non-structural protein NS3/Small envelope protein E	9	62	1	74	75	32.2	8.20E-08
			Insignificant	EVC2_like	Ellis van Creveld protein 2 like protein	53	99	1	69	429	13.5	2.10E-02
			Insignificant	FAM163	FAM163 family	13	36	8	66	163	11.8	2.40E-01
2	Severe acute respiratory syndrome-related coronavirus	APO40581.1	**Significant**	NS3_envE	Non-structural protein NS3/Small envelope protein E	9	66	1	76	75	32.4	7.40E-08
			Insignificant	EVC2_like	Ellis van Creveld protein 2 like protein	53	100	1	71	429	14	1.50E-02
			Insignificant	FAM163	FAM163 family	13	36	8	66	163	11.7	2.70E-01
3	Coronavirus BtRl-BetaCoV/SC2018	QDF43816.1	**Significant**	NS3_envE	Non-structural protein NS3/Small envelope protein E	9	64	1	76	75	28	1.70E-06
			Insignificant	EVC2_like	Ellis van Creveld protein 2 like protein	53	100	1	72	429	14.8	8.50E-03
4	Rhinolophus affinis coronavirus	AHX37560.1	**Significant**	NS3_envE	Non-structural protein NS3/Small envelope protein E	9	71	1	76	75	32.6	6.20E-08
			Insignificant	EVC2_like	Ellis van Creveld protein 2 like protein	53	100	1	71	429	14.1	1.40E-02
5	SARS coronavirus GD01	AAP51230.1	**Significant**	NS3_envE	Non-structural protein NS3/Small envelop	9	68	1	76	75	32.3	7.80E-08
			Insignificant	EVC2_like	Ellis van Creveld protein 2 like protein	53	100	1	73	429	13.9	1.60E-02
6	Severe acute respiratory syndrome coronavirus 2	YP_009724392.1	**Significant**	NS3_envE	Non-structural protein NS3/Small envelope protein E	9	62	1	74	75	32.2	8.20E-08
			Insignificant	EVC2_like	Ellis van Creveld protein 2 like protein	53	99	1	69	429	13.5	2.10E-02
			Insignificant	FAM163	FAM163 family	13	36	8	66	163	11.8	2.40E-01

Structure Assessment

**Structure assessment of SARSCoV-1-E Protein**		
MolProbity Score	1.66	
Clash Score	7.37	
Ramachandran Favoured	0.973	
Ramachandran Outliers	0	
Rotamer Outliers	0.0143	A76 VAL
C-Beta Deviations	1	A58 ILE
Bad Bonds	0 / 602	
Bad Angles	9 / 822	A56 PHE, A20 PHE, A4 PHE, A26 PHE, A42 TYR, A32 ALA, A19 LEU, (A32 ALA-A33 ILE)
**Structure assessment of SARS-CoV-2-E Protein**		
MolProbity Score	1.33	
Clash Score	4.12	
Ramachandran Favoured	0.9726	
Ramachandran Outliers	0.0137	A69 ARG
Rotamer Outliers	0	
C-Beta Deviations	1	A43 CYS
Bad Bonds	0 / 598	
Bad Angles	8 / 817	A4 PHE, A20 PHE, A45 ASN, A69 ARG, (A70 VAL-A71 PRO), A12 LEU, (A68 SER-A69 ARG), A32 ALA
