# Supplementary material for: Microsecond molecular dynamics simulations revealed the inhibitory potency of amiloride analogs against SARS-CoV-2 E viroporin
Source: Genomics Inform. 2021 Dec 31;19(4):e48. doi: 10.5808/gi.21040 (PMC8752979; doi:10.5808/gi.21040)
Supplement: Supplementary Data S2. [file gi-21040suppl2.pdf]

Supplementary file: 2. Physicochemical and pharmacokinetic properties of HMA and 3A5NP2C

Physicochemical Properties

Drug Compound	PubChem CID	Formula	MW	#Heavy atoms	#Aromatic heavy atoms	Fraction Csp3	#Rotatable bonds	#H-bond acceptors	#H-bond donors	Molar Refractivity	Topological polar surface area (TPSA)
5-(N,N-Hexamethylene)amiloride	1794	$C_{12}H_{18}ClN_7O$	311.77	21	6	0.5	3	4	3	85	136.51
3-amino-5-(azepan-1-yl)-N-(diaminomethylidene)-6-pyrimidin-5-ylpyrazine-2-carboxamide	137348787	$C_{16}H_{21}N_9O$	355.4	26	12	0.38	4	6	3	101.01	162.29

Lipophilicity

iLOGP	XLOGP3	WLOGP	MLOGP	Silicos-IT Log P	Consensus Log P
1.75	1.3	0.13	0.79	0.11	0.82
2.05	0.29	-0.06	-0.09	-0.09	0.42

Water Solubility

ESOL Log S	ESOL Solubility (mg/ml)	ESOL Solubility (mol/l)	ESOL Class	Ali Log S	Ali Solubility (mg/ml)	Ali Solubility (mol/l)	Ali Class	Silicos-IT LogSw	Silicos-IT Solubility (mg/ml)	Silicos-IT Solubility (mol/l)	Silicos-IT class
-2.61	7.73E-01	2.48E-03	Soluble	-3.77	5.33E-02	1.71E-04	Soluble	-2.22	1.87E+00	6.01E-03	Soluble
-2.3	1.77E+00	4.97E-03	Soluble	-3.26	1.95E-01	5.49E-04	Soluble	-3.35	1.58E-01	4.46E-04	Soluble

Pharmacokinetics

GI absorption	BBB permeant	Pgp substrate	CYP1A2 inhibitor	CYP2C19 inhibitor	CYP2C9 inhibitor	CYP2D6 inhibitor	CYP3A4 inhibitor	log Kp (cm/s)
High	No	Yes	Yes	Yes	No	No	No	-7.28
Low	No	Yes	No	No	No	No	No	-8.26

Druglikeness

Lipinski #violations	Ghose #violations	Veber #violations	Egan #violations	Muegge #violations	Bioavailability Score
0	0	0	1	0	0.55
0	0	1	1	1	0.55

Medicinal Chemistry

PAINS #alerts	Brenk #alerts	Leadlikeness #violations	Synthetic Accessibility
0	2	0	3.27
0	2	1	3.57
